# The cultural adaptation of Western trauma interventions for Indigenous peoples experiencing trauma: an environmental scan of Turtle Island

**DOI:** 10.3389/fpsyg.2026.1927776

**Published:** 2026-10-29

**Authors:** Sophia Gran-Ruaz, Mirana Rambelo, Monnica T. Williams

**Affiliations:** School of Psychology, University of Ottawa, Ottawa, ON, Canada

**Keywords:** cultural adaptation, cultural safety, environmental scan, indigenous peoples, trauma

## Abstract

**Background:**

Experiences of oppression and genocide towards Indigenous peoples have contributed to severe trauma symptoms. Yet, for Indigenous individuals seeking trauma-related help in Western clinical settings, most therapists lack the culturally relevant tools to meet the needs of Indigenous peoples soliciting help.

**Objective:**

As Western-informed trauma interventions are developed for use broadly, this article reviews how said interventions are currently adapted by key parties to be safe, effective, and relevant for Indigenous individuals specifically.

**Method:**

Source eligibility included: at least one Indigenous author or an Indigenous community/organization as collaborator; a Western-based trauma intervention adapted for use with Indigenous individuals; and a focus on the populations of Turtle Island (so called “North America”). Western and Indigenous databases, as well as non-academic literature, were searched.

**Results:**

A summary of current adaptation practices is presented in three phases: (i) planning (selecting an appropriate therapy, consultation and collaboration with communities/experts); (ii) adaptation (surface and deep level); and (iii) implementation (assessing client fit, evaluating feasibility and effectiveness).

**Conclusion:**

Key parties described their adaptation processes in detail, but there appears to be little consensus on how to go about this work. As such, the synthesis of formalized guidelines or Wise Practices could be of great benefit.

## 1 Background

Experiences of past and present oppression and genocide towards the Indigenous peoples of Turtle Island have contributed to severe symptoms of trauma (de la Salle et al., 2022). Yet, for Indigenous peoples experiencing trauma seeking help in Western clinical settings, Settler^1^ and other non-Indigenous mental health providers often lack the culturally relevant tools and trauma interventions to meet their unique needs (Anderson, 2019; Herron and Venner, 2023; Hyett et al., 2018; Panofsky et al., 2021). As Western-informed trauma interventions are developed for use with clients more broadly, the present authors consider how such interventions may be adapted to be safe, effective, and culturally relevant for Indigenous peoples soliciting help specifically. The current article is the first in a multi-article series which scans the North American landscape to better understand what clinicians and researchers are currently doing to culturally adapt Western trauma treatments for use with this population. That is, without evaluating or assigning worth, what cultural adaptation practices have been tried and tested? In so doing, this article aims to serve as a preliminary step in answering a call from Indigenous health and trauma knowledge holders, for those key parties striving to be allies to engage in more applied trauma research and offer more culturally relevant services.

### 1.1 Indigenous communities of Turtle Island and trauma

At the time of European arrival in 1492, what many Nations refer to as Turtle Island (i.e., North America) was already home to millions of Indigenous peoples with over 500 distinct groups (Waldram et al., 2006), over 50 distinct languages (Waldram et al., 2006), and thousands of independent bands and tribes (Waldram et al., 2006). Moreover, many of these groups were strong and healthy, with fully functioning and highly complex societies in possession of thriving governance systems, traditional laws and economies, technologies (calendars, agriculture, water management), traditions, spiritualities, medicines, epistemologies, ontologies, and much more. After hundreds of years of discrimination, oppression, and genocide at the hands of Settlers, large disparities are seen in the mental health of Indigenous peoples today compared to their non-Indigenous counterparts (Gone et al., 2019)^2^. More specifically, Indigenous peoples in North America present with disproportionate rates of anxiety and depression (Bellamy and Hardy, 2015a, 2015b; Hop Wo et al., 2020), sleep disturbances (Chapman et al., 2013), substance and alcohol misuse (Firestone et al., 2015; Patterson-Silver Wolf et al., 2015), and suicide behaviors (Kirmayer et al., 2007; Kumar et al., 2019). Despite evidence of impacts across several wellness domains, the vast majority of Indigenous mental health research focuses on the subject of trauma.

The Indigenous peoples of Turtle Island experience traumatic stress through two predominant pathways: historical and contemporary traumas (Gameon and Skewes, 2019). The first trauma type has many names including intergenerational trauma, collective trauma, soul wound, and historical loss, just to name a few (Fast and Collin-Vézina, 2020; Mitchell et al., 2019). Despite the differing labels, most characterize such traumas as colonial injuries done to Indigenous peoples by Settler populations resulting in intense loss, grief, and pain (Hartmann and Gone, 2014). Moreover, these cumulative effects are felt collectively by entire Indigenous communities and across many generations (Hartmann and Gone, 2014; Kirmayer et al., 2014). Consider for example the collective suffering felt by many of today’s Indigenous peoples from the Indian Residential School system (Bombay et al., 2014), the forced relocation of ancestors from traditional lands (Walls and Whitbeck, 2012), the forced or coerced sterilization of Indigenous women (Leason, 2021), or the past (and in many ways still present) suppression/illegalization of spiritual and cultural practices (Edwards, 2020). The effects of these and other forms of colonial violence are transmitted culturally, and perhaps also biologically (Rogers-LaVanne et al., 2023; Schafte and Bruna, 2023), regardless of if Indigenous individuals lived these events themselves.

These historical traumas are compounded by and contribute to current contemporary traumas. More specifically, Indigenous peoples are at a greater risk of experiencing community violence, interpersonal violence, and childhood abuse (Bellamy and Hardy, 2015c; Spillane et al., 2022). As such, Indigenous peoples are at great risk of developing severe trauma responses like posttraumatic stress disorder (PTSD) (Williams et al., 2022), which is a disorder characterized by intrusion symptoms (flashbacks, recurrent nightmares), avoidance behaviors, negative changes in cognition and mood (distorted blame, embarrassment, isolation, dysphoria) as well as alterations in arousal and reactivity (self-destructive behaviors, hypervigilance, sleep issues). Naturally, these symptoms lead to significant impairment in one’s relationships and occupations. Due to their increased likelihood of contemporary trauma exposure, some Indigenous members are also at greater risk of developing complex-PTSD (Gameon and Skewes, 2019; O’Neill et al., 2018; Radford et al., 2022), a disorder seen in individuals with a history of prolonged, chronic, and repeated traumatic stress occurring in their formative childhood years. Moreover, racial trauma, or the cumulative emotional and spiritual injury experienced as a result of the racism and discrimination across the lifespan, is also likely pervasive among Indigenous populations (Williams et al., 2022; Cénat, 2023; Environics Institute, 2010).

### 1.2 A call for culturally relevant trauma interventions

Indigenous peoples are among the most studied populations in the world, yet Indigenous scholars criticize that most of Indigenous health research is descriptive in nature, with very little being interventional or applied (Anderson, 2019; Gone and Trimble, 2012). Further, the few interventional studies undertaken “fail to meet acceptable standards of scientific rigour or quality (Anderson, 2019, p. 2).” Others also point to a lack of openness among non-Indigenous researchers to traditional Indigenous healing practices and/or willingness to collaborate with Indigenous peoples when undertaking such work (Hyett et al., 2018). As a result, Indigenous health research, and by extension, its outputs, seldom meet Indigenous needs or align with Indigenous values. Therefore, Indigenous and non-Indigenous knowledge holders in Indigenous health push decision makers to develop systems and services that are holistic, culturally rooted, and that better understand the mental health realities of Indigenous peoples (Montesanti et al., 2022).

Within trauma intervention research specifically, Panofsky et al. (2021) calls on researchers and clinicians to: engage in research representing the heterogeneity of Indigenous communities; use participatory approaches throughout the varied stages of the research, analysis, and dissemination process; as well as conduct trauma intervention research that is inclusive, engaged, empowering, and culturally relevant. Moreover, as Indigenous scholars Duran and Firehammer (2017) explain, “unless theory, practice, and research are deeply rooted in the life-world metaphor of the culture, effectiveness will be limited at best, and more trauma will occur at worst (p. 122).” Therefore, the use of Western-created trauma interventions, absent attempts to make them more culturally relevant, may in fact cause more trauma than they alleviate in Indigenous sufferers.

This sentiment is echoed in the limited research that considers Indigenous peoples’ perceptions of and barriers to accessing mental health services within Western settings. For instance, in a Canada-wide survey of the general population (Faber et al., 2023), 50% of Indigenous respondents reported at least one past negative experience accessing mental health services. There are many reasons as to why that may be and, to investigate, Smye et al. (2023) undertook interviews and focus groups with Indigenous clients accessing five community-based mental health care agencies in Canadian urban centers. Findings from these conversations included that Western services absent cultural attunement can recreate trauma and serve as “an extension of the marginalizing experiences that many participants described in everyday interactions outside of healthcare (p. 7).” Moreover, current treatment paradigms fail to understand and attend to the structural inequities and structural violence Indigenous peoples face. Nor do Western mental health systems’ emphasis on individualized biomedical treatments align with many Indigenous conceptualizations of wellness, which value holistic, relational, and collective approaches to healing, including connections among mind, body, spirit, family, community, culture, and land (Coe et al., 2025). In a separate integrative review of 41 studies exploring barriers to Indigenous peoples accessing mental health care (Goetz et al., 2023), many Indigenous peoples found Western evidence-based treatment lacking as it seldom included cultural considerations. Further, a 2021 survey of American mental health professionals serving Indigenous populations (Knowlton and Lafavour, 2021) found that 88% of participating practitioners also believed the current standard of evidence-based practice failed to provide culturally informed care options.

All-in-all there is a need for culturally relevant trauma interventions to support Indigenous healing. According to scholars, the recommended first-line way to ensure cultural relevancy are trauma interventions that are *culturally grounded* (Panofsky et al., 2021; Gameon and Skewes, 2019; Linklater, 2014). That is those original interventions that are created by Indigenous communities using a ground up approach, for use in said communities, and deeply rooted in Indigenous values, behaviors, norms, and worldviews (Gameon and Skewes, 2019; Okamoto et al., 2014). The unfortunate reality is that many Indigenous peoples experiencing trauma, fall through the cracks and do not have access to such first-line solutions (Kirmayer et al., 2021). For one, there are Indigenous communities who do not have the capacity to offer culturally grounded trauma services to all the members that need it. There are Indigenous folks, who for many reasons, may not have ready access to or are disconnected from their communities, and thus require culturally relevant services elsewhere. There are also those Indigenous individuals who are acculturated in some degree to both Indigenous and Western ways of knowing/being. These peoples experiencing trauma either fail to receive the care they need or try to access a Western health setting, where they are often served by non-Indigenous clinicians, and with culturally inappropriate techniques.

It is such circumstances that lead to a possible second-line approach that is used by some communities but can potentially also be used in Western clinical settings – *culturally adapted* services. With culturally adapted trauma interventions, a form of cultural relevancy can be achieved by altering an existing and empirically-based Western-created intervention to meet the specific cultural needs of Indigenous peoples (Breckin et al., 2026). Given it is a process of tweaking and not starting-from-scratch, culturally adapted techniques are often more time and cost-effective (Okamoto et al., 2014). Further, several meta-analyses have spoken to their possible effectiveness. An initial meta-analysis by Griner and Smith (2006) reviewed culturally adapted mental health interventions in ethnic minority groups more broadly and found a moderate positive effect in treatment outcomes (*d* = 0.45 across 76 studies). There have been several similar meta-analyses since, which enabled Rathod et al. (2018) to summarize many reviews. Rathod noted effect sizes between 0.23 and 0.75, with majority of culturally adapted mental health interventions reporting moderate to large effect sizes.

### 1.3 How to culturally adapt interventions

There are no agreed upon best practice guidelines for culturally adapting mental health interventions for different ethnic groups more broadly, let alone for trauma interventions and/or Indigenous peoples. However, Bernal et al. (1995) have proposed an early cultural adaptation framework known as the *Ecological Validity Model*. Based on Bronfenbrenner’s ecological systems theory, said model proposes eight domains for possible adaptation consideration. Domains include language (culturally appropriate and responsive language), metaphors (symbols, sayings, and concepts), content (cultural knowledge about values, customs, and traditions), concepts (culturally and contextually agreeable treatment constructs), goals (positive and adaptive treatment targets), persons (delivery by those of a similar ethnoracial background or with similar cultural values), methods (procedures used to reach treatment goals), and context [psychological, developmental, social, economic and political context; (Bernal et al., 1995; Sit et al., 2020)].

Another pertinent model of cultural adaptation is the *Cultural Sensitivity Model* (Resnicow et al., 1999) that remains in wide use today. Said framework denotes that there are two levels for informing cultural adaptations. The first level is known as the *surface* level. Surface level adaptations aim to “match materials (illustrations, language), as well as channels and settings for treatment delivery to observable characteristics of the target population (Heim and Kohrt, 2019, p. 5).” Examples include making adjustments to the language of the original intervention, either by translating the intervention into the target groups’ language or using more culturally appropriate phrases and metaphors. Surface adaptation may also look like creating culturally appropriate visuals to replace those in the original program. Other surface-level changes may include using places, food, music, people, or clothing familiar to the target group. Conversely, *deep* level adaptations consider how “cultural, social, psychological, environmental, and historical factors influence health behaviors differently across racial/ethnic populations (Resnicow et al., 1999).” As such, Resnicow et al. (1999) explains that surface level adaptation serves to increase the receptivity and acceptance of an intervention’s messages, while deep level adaptation conveys salience. A word of caution: although deep level adaptations touching on the cultural values of a target group can be beneficial, one must be mindful that such changes may inadvertently alter core therapeutic change mechanisms in the original intervention, making it less effective (Okamoto et al., 2014; Castro et al., 2010).

### 1.4 Study purpose

While the old frameworks remain helpful, a lack of accessible and concrete cultural adaptation guidelines generally, but also unique to Indigenous populations, may leave some striving to be allies unsure of how to support Indigenous peoples experiencing trauma. Speaking anecdotally of discussions within my (first author’s) personal network and my own experience, many non-Indigenous mental health providers and researchers want to support processes of truth and reconciliation but lack the knowledge or resources necessary to do so in a culturally safe, effective, and sensitive way (Hemmings and Evans, 2018). Such clinicians and researchers striving to be allies could, therefore, greatly benefit from a set of “Wise Practices” or recommendations to ensure trauma interventions are safe and appropriate for use with Indigenous persons soliciting Western services.

With this in mind, the authors undertake the development of a series with three main goals. Most obviously, the team will strive to synthesize Wise Practice guidelines (Wesley-Esquimaux and Calliou, 2010) for the cultural adaptation of Western-created trauma interventions for use with Indigenous clients in clinical settings. Second, this series aims to model how to go about applying said Wise Practices by discussing the possible cultural adaptation process of an existing racial trauma intervention. And finally, as a team made up of non-Indigenous researchers, the authors aim to model respectful, collaborative, and reciprocal relationships between Indigenous and non-Indigenous partners and thus allyship behavior^3^. All-in-all, this series asks [i] What, if any, Wise Practices do Indigenous trauma knowledge holders identify for adapting Western trauma treatments for use with Indigenous clients? and [ii] How could said Wise Practices be applied to a real-world trauma intervention example?

However, as the first article of this multipart series, the team explores absent formal guidelines, what have researchers and clinicians been doing in their attempts to culturally adapt trauma treatments for Indigenous individuals and communities in distress? As such, this first article acts as an environmental scan of sorts: a review of the current landscape of cultural adaptation practices targeting Indigenous peoples experiencing trauma. At this stage, the authors do not attempt in any way to appraise, positively or negatively, what has been done by these key parties to date. This appraisal comes in subsequent parts of the series.

### 1.5 Positionality

When undertaking research with Indigenous peoples, it is essential we position ourselves in the research (American Psychological Association, 2023; Kovach, 2021; Wilson, 2008). As Renee Linklater states “First, we write our own stories and share our position in the world before we write about the world. […] we have to come to terms with who we are and how we come to do the work we do (Linklater, 2014, p. 11).” As such, the primary author identifies as a first generation Canadian, of Austrian and Scottish ancestry. Said author grew up in a time and school system where Indigenous peoples, their histories, ways of knowing and being, and survivance were seldom discussed. Her allyship journey began through work with people experiencing homelessness, where relationships with Indigenous clients using the shelter system exposed her more directly to histories of oppression, attempted assimilation, and trauma arising from ongoing colonial harms. Since then, she has pursued further learning through education, training, research, and sustained involvement with members/organizations of her local urban Indigenous community. Presently she recognizes that this learning will remain ongoing and strives to approach this work with humility. She also seeks to use her skills, resources, and privilege in ways that are respectful and of value to the communities and individuals from whom she has learned so much.

The second author served as a research assistant on this project. As a child of immigrants from Madagascar raised in suburban middle-class environments, a member of the queer community, and a researcher engaging with marginalized communities, she brings multiple perspectives to her work. Her collaborative research with Métis colleagues and fieldwork in Brazilian communities have deepened her critical awareness of power dynamics in academic research and health care systems. Shaped by the colonial histories that affected her family’s migration and Indigenous Peoples broadly, she approaches this research with a deep commitment to ethical engagement and centering Indigenous voices, knowledge systems, and perspectives.

The senior author is a Canada Research Chair at a major urban university, a registered clinical psychologist, and an African American woman. She has published over 200 peer-reviewed articles, with a focus on trauma-related conditions and cultural differences, including articles about therapeutic best practices. She is deeply concerned about the ongoing oppression experienced by Indigenous people in Canada and the layers of traumatization that continue to impact these communities.

## 2 Method

### 2.1 Search strategy

This work was undertaken as an environmental scan of the literature rather than a formal scoping or systematic review. The aim was to broadly map the extent and nature of cultural adaptation activities, including the range of processes undertaken. This environmental scan in no way seeks to evaluate the effectiveness of these adaptation efforts or determine whether they align with preferred practices or approaches. Nevertheless, a rigorous search strategy was used to support a sufficiently broad capture of the relevant literature, not to achieve exhaustive identification of all available evidence.

With the aforementioned in mind, several databases were included in this environmental scan including Western (PubMed, PsycINFO, Social Services Abstracts [Sociological Abstracts + Sociology Database], Web of Science, Google Scholar) as well as Indigenous-specific databases (Native Health Database, Bibliography of Indigenous Peoples in North America, Indigenous Studies Portal Research Tool). Non-academic literature was also accessed (websites, technical reports, YouTube, etc.). For Google Scholar and non-academic sources, the search was stopped when 100 consecutive hits yielded no additional relevant articles. Academic and non-academic sources were initially searched in the Summer and Fall of 2024. As part of the publication revision process, the scan was updated through a supplementary search of two Western databases (Google Scholar and PsycINFO) and relevant grey literature to identify more recent work.

Four groups of keywords were used:

- *Population…* (Indigenous OR Aboriginal* OR “First Nation*” OR “First People*” OR Inuit* OR Metis OR Native OR Amerind* OR “Indians of North America” OR Indians) AND
- *Trauma…* (“racial trauma” OR “race-based traumatic stress” OR “colonial trauma” OR “intergenerational trauma” OR “historical trauma” OR “cultural trauma” OR “post-traumatic stress disorder” OR “psychological trauma” OR “soul wound” OR “substance use” OR “addiction” OR “alcoholism”) AND
- *Treatment…* (intervention OR treatment OR “healing” OR “land-based” OR “wholistic” OR “holistic” OR service* OR therap* OR counsel*) AND
- *Adaption…* (“adapt*” OR “two-eye*” OR “integrat*” OR “blend*” OR “tailor*”)

A few notes of clarification on our operational definition of “trauma intervention.” In defining trauma interventions, we approached the term more broadly than most Western mental health stakeholders and using a decolonial lens. Indigenous scholarship does not limit trauma to a single traumatic or stressful event, nor a neatly packaged suite of symptoms (Fast and Collin-Vézina, 2020; Mitchell et al., 2019; Gone, 2013). Trauma can be understood to stem from an individual’s experiences but also through the experiences of loved ones, their community, and even by long passed ancestors (see *Background* for additional details). Symptoms can look like the typical posttraumatic intrusion memories, avoidance, negative changes in thinking and mood, and changes in physical and emotional reactions, but they can also look like alcohol and substance use, depression, anxiety, suicidal thinking, etc. (Brave Heart, 1998; Heart, 2003). With that said, logistically some manageable parameters needed to be set for this environmental scan. As such, interventions targeting PTSD, race-based traumatic stress, collective trauma, intergenerational trauma, alcohol and substance use were included. Further, given health promotion and prevention intervention programs often focus *proactively* to reduce future traumatic stress risk, said trauma interventions were also excluded from this review.

Material was eligible for inclusion if it was (i) written in English; (ii) had one or more authors who self-identified as Indigenous or it was explicitly created in collaboration with an Indigenous community/organization; (iii) described a Western-based trauma intervention that was adapted for use with Indigenous peoples; and (iv) focused on Indigenous populations based on Turtle Island (i.e., Canada or the United States). On inclusion criterion iii, while this environmental scan does not wish to assign valence to what key parties are doing in their attempts to culturally adapt Western trauma interventions, the authors do not want to center potentially harmful practices. Further, while there is no set of recommended practice guidelines for the adaptation of trauma interventions, there are many longstanding general research practice guidelines that hold the principle of Indigenous consultation as a central tenant – “nothing about us, without us” (Castellano and Reading, 2010). As such, inclusion of Indigenous communities or partners in the adaptation process was seen as a bare minimum allied practice required for inclusion in this scan. Peer reviewed searches were restricted to the abstract level. No publication date limit was imposed.

Finally, the authors also supplemented database search outputs with systematic review and scoping review citations. That is, any related articles not previously captured by the aforementioned search terms, but present in related review papers (e.g., Panofsky et al., 2021; Kowatch et al., 2019; Richer and Roddy, 2023), were considered for inclusion in this environmental scan.

Of note, the search strategy described here-in was reviewed and approved by two speciality librarians at the PI’s home institution.

## 3 Findings

In completion of this environmental scan, it was learned that there have been many previous attempts within Turtle Island to adapt Western-originating trauma interventions for use with Indigenous peoples. The earliest attempts originating from the late 1980s. Broadly speaking, most adapted interventions were created with the intention they be used within Indigenous communities or community-adjacent towns (e.g., Bedard-Gilligan et al., 2022; Horn et al., 2008; Pearson et al., 2019; Venner et al., 2016). There were fewer, albeit still some, examples of adaptation for use within urban clinical settings (e.g., Dickerson et al., 2016; Edwards, 2003; Reeves and Stewart, 2014; Tsosie et al., 2011). Moreover, there were examples of adaptation interventions targeting a specific nation or tribe (e.g., Kelley et al., 2017), but also those aimed at supporting on a pan-Indigenous level [most commonly in urban settings, when several diverse band and tribe affiliations were present; (e.g., Saylors, 2003)]. Some “key parties,” that is individuals involved in the adaptation process, attempted balancing both adapting for as widespread a group as possible while also appreciating the great heterogeneity found between varied Indigenous groups and peoples (e.g., Bedard-Gilligan et al., 2022; Dickerson et al., 2016).

For easy accessibility the authors chose to present the findings from the environmental scan chronologically. That is what was described as happening before (i.e., planning), during (i.e., active adaptation), and after (i.e., implementation) the attempted adaptation of a Western trauma intervention for use with Indigenous peoples experiencing trauma. See Supplementary Table A for a high-level overview of the cultural adaptation efforts discussed in varying levels of depth throughout this environmental scan.

### 3.1 The planning stage

#### 3.1.1 Expert and community engagement

Much of the literature encountered for the environmental scan described needing a multidisciplinary team to achieve adaptation goals. Most discussed was the use of “community review committees” (CRCs). Otherwise referred to as “community boards” (e.g., Hirchak et al., 2023), “advisory groups” (e.g., Marsh et al., 2015b), “tribal advisory boards” (e.g., Pearson et al., 2019), “steering committees” (e.g., Novins et al., 2012), among other names, said groups comprised of a collective of experts and/or key parties impacted by the outcomes of one’s project. All within were tasked with providing focused advice and recommendations on the project in question. In consideration of the specific elements of the project, CRC guidance was described as supporting the education of research teams on population/community cultural issues and values (e.g., Horn et al., 2008; Walker et al., 2023), selection of a suitable therapy (e.g., Bedard-Gilligan et al., 2022), the adaptation process itself (e.g., Bedard-Gilligan et al., 2022; Hirchak et al., 2023; Novins et al., 2012; McDonell et al., 2016), selection of appropriate measures and outcomes (e.g., Goodkind et al., 2010), as well as promoting outcomes at practice and policy levels (e.g., Horn et al., 2008). And sometimes CRCs were deemed necessary for building/facilitating relationships and partnerships between the research team and local community (e.g., Hirchak et al., 2023), and building/strengthening the alliance with non-Indigenous therapists (e.g., Linnaranta et al., 2024).

CRCs were often made up of diverse Indigenous and non-Indigenous experts. Those key parties who described their CRC composition in detail commonly spoke to the inclusion of Elders or traditional knowledge keepers (e.g., Bedard-Gilligan et al., 2022; Kelley et al., 2017; Hirchak et al., 2023; Marsh et al., 2015b; Novins et al., 2012), service program providers (e.g., Bedard-Gilligan et al., 2022; Hirchak et al., 2023; McDonell et al., 2016), traditional healers (e.g., Bedard-Gilligan et al., 2022; Hirchak et al., 2023; Marsh et al., 2015b), and clients/caregivers (e.g., Hirchak et al., 2023; McDonell et al., 2016; Burduli et al., 2018; D’Amico et al., 2020; Motta-Ochoa et al., 2026). Others spoke to the inclusion of community members (e.g., Venner et al., 2008), tribal leaders (e.g., Bedard-Gilligan et al., 2022), and tribal linguists (e.g., Bedard-Gilligan et al., 2022; Marsh et al., 2015b). In the case of adapted treatments for substance use disorders, physicians were often included as experts (e.g., Hirchak et al., 2023; Marsh et al., 2015b). Some key parties recruited experts from across a large geographical area to increase generalizability of the adaptation (e.g., Hirchak et al., 2023). Others recruited more locally so as to ensure local band/tribal relevance (e.g., Marsh et al., 2015a). Some went so far as to manage multiple CRCs simultaneously when working with more than one community (e.g., Dickerson et al., 2016). Others observed that communities (and thus resulting CRCs) identified themselves more fluidly, either geographically, or using other factors (e.g., Horn et al., 2008). For instance, perhaps community groups were defined by shared common agendas, objectives, interests and needs, experiences of Settler colonialism, social ties, or commitments to action (Horn et al., 2008).

In terms of how said CRCs were engaged, some key parties opted for a single intensive meeting, while others preferred prolonged and very regular contact. For example, McDonell et al. (2016) adapted a contingency management intervention for use with Indigenous clients experiencing alcohol use difficulties. As an element of the adaptation process, the research team assembled a CRC composed of five community members. The CRC attended a two-day meeting where they discussed the study design, procedures, and timeline, and any recommended changes in detail. McDonell also described having subsequent meetings with the CRC as needed. Similarly, in adapting an intervention for the delivery of opioid use disorder medications, Hirchak et al. (2023) held their own two-day retreat with their CRC. Meeting topics included “understanding the etiology of addiction from an Indigenous perspective; concepts of wellness, pain, and recovery; best practices for engagement, treatment delivery, and recovery; and integration of traditional practices into healthcare settings (p. 175).” Moreover, Hirchak used a World Café qualitative facilitation method and many breakout activities to facilitate meaningful discussion. Walker et al. (2023) opted for a mix of in-person meetings and regular written summary reports with their CRC. Conversely, Marsh et al. (2015a) described taking a much slower community engagement and relationship building approach. Prior to developing her CRC, Marsh spent a year meeting with Elders in her nearby Indigenous community on a weekly basis, using the time to understand more about their community, realities, as well as ethics and local protocols. Eventually Marsh established a CRC, and they convened monthly.

#### 3.1.2 Identifying, understanding, and describing needs and realities

Within the academic and non-academic discourse, it appeared to the writers that most adaptation project research teams felt assured they had a well-developed understanding of the needs and realities of the Indigenous clients and communities they were trying to support. That is, they were confident in understanding the origins and impacts of the trauma response, and the challenges experienced by said communities. Or at the very least, they felt they could lean on the expertise of their CRCs to fill in any knowledge gaps that arose over the life of their project and as such, were ready to “hit the ground running”.

In contrast, a smaller number of key parties discussed engaging in a great deal of work to educate themselves on their respective issue prior to initiating the adaptation process. For some, *needs assessments* or systematic evaluations for identifying community priorities were first undertaken (e.g., Bedard-Gilligan et al., 2022). Others conducted interviews with a small sample of prospective future clients and/or service providers to better understand the problem, barriers to recovery, existing coping mechanisms/healing approaches, and cultural needs, among other things (e.g., Ashby et al., 1988; Martin, 2013; Campbell et al., 2023; Hirchak et al., 2026). In the case of Horn et al.’s adaptation of a tobacco cessation program for Indigenous youth (Horn et al., 2008), key parties went a step further to explore the challenges associated with tobacco use, as well as its functional value within the communities supported. In so doing, they learned that not only is tobacco sacred, but said communities are heavily reliant on the tobacco economy. Therefore, the adapted program shifted in focus from targeting tobacco and tobacco use more broadly, to targeting problematic tobacco addiction.

#### 3.1.3 Therapy selection

Western interventions selected for adaptation included cognitive behavioral therapies (e.g., Martin, 2013; Bigfoot and Schmidt, 2010), cognitive processing therapy (e.g., Pearson et al., 2019), a community reinforcement approach (e.g., Venner et al., 2016), contingency management (e.g., Novins et al., 2012; McDonell et al., 2016), dialectical behavior therapy (e.g., Beckstead et al., 2015), interpersonal psychotherapy (e.g., Brave Heart et al., 2020), medication-assisted therapy (e.g., Hirchak et al., 2023), motivational interviewing (e.g., Venner et al., 2008; Dionne et al., 2009), and narrative exposure therapy (e.g., Bedard-Gilligan et al., 2022), among others. Nevertheless, some made clear that not all Western trauma therapies are created equally. That is, in the selection of an appropriate intervention the literature discussed what made some Western trauma treatments more appropriate for adapted use with Indigenous peoples than others. More specifically, oral traditions are seen as a key stone of knowledge transfer within Indigenous communities, and storytelling is how this knowledge is shared. Some go so far as to characterize storytelling as a “medicine” in itself (Martin, 2013; Fox and Heitkamp, 2022). Therefore, Western interventions with large existing storytelling components were deemed more readily adaptable (e.g., Bigfoot and Schmidt, 2010). For example, Bedard-Gilligan et al. (2022) chose to adapt narrative exposure therapy (NET). NET helps trauma survivors by working to build a consistent and chronological narrative of their lives, incorporating (in detail) any past traumatic and stressful events. In so doing, individuals are exposed and habituated to, as well as make meaning of, any impactful traumatic experiences. Bedard-Gilligan chose this type of trauma therapy in concert with members of an Indigenous community in the United States due to NET’s techniques aligning heavily with Indigenous traditional practices like storytelling, and tactile symbolism (Bedard-Gilligan et al., 2022). In another adaptation initiative, Pearson et al. (2020) also saw NET as an especially viable adaptation option for the aforementioned reason, and also on account that it allowed individuals to address multiple traumatic experiences, as opposed to being anchored in/focusing on a single index trauma.

Others selected Western trauma treatments with more of a spiritual basis, and/or those deemed as more strengths-based and empowering. For instance, in adaptation of an intervention for treatment of both substance use and intergenerational trauma, Marsh et al. (2015a) chose *Seeking Safety*. As a component of therapy, the original *Seeking Safety* protocol encouraged spiritual discussions, and sought to heal the mind, body, and spirit collectively. Further, the original model was deemed to be “positive, strengths-based, respectful, supportive, and collaborative (p. 7),” therefore, aligning with many Indigenous healing methods and values. Some also spoke to valuing Western therapies with a built-in group component. More specifically, said key parties prized group therapy’s natural similarity with Indigenous sharing circles and ability to foster/strengthen a sense of community and connection with others (e.g., Marsh et al., 2015a,b; Heilbron and Gutrman, 2000; Marsh et al., 2016).

Alternatively, a few key parties raised concerns with more cognitive-based approaches serving as starting points for the adaptation process. In his seminal work, Duran (2006) alluded to how cognitive therapy approaches can sometimes feel as though they conflict with Indigenous people’s worldviews as they focus on only one aspect of the person and wellness, cognitions, ignoring other key elements. And in Linnaranta et al.’s (2024) work to adapt cognitive behavioral psychotherapy and virtual reality exposures for Inuit in Nunavik, advisory groups shared concerns about certain cognitive elements. That is, cognitive restructuring techniques were deemed potentially culturally harmful and colonialist. As one CRC member explained “Putting thoughts and ideas in perspective, testing alternative thoughts, I think it can be culturally harmful because it is invalidating the person’s experience and where they come from, and there is a reason why they are having these experiences, there is a reason why they are having these coping strategies, and it need[s] to be validated (p. 5)”.

### 3.2 The adaptation stage

#### 3.2.1 Surface level adaptations

As described above, surface level adaptations are changes made which help clients be better able to connect with intervention messaging, without altering core aspects of the intervention. Of note, some assert that simply adding in some cultural elements (surface level changes) without broader engagement of deep level adaptation is “tokenistic” (Roy et al., 2015, p.74, 93).

##### 3.2.1.1 Visuals

As part of the adaptation process, some key parties described altering the visuals of the original trauma intervention’s manuals and other program materials (e.g., Bedard-Gilligan et al., 2022; Bigfoot and Schmidt, 2010). For select teams, this meant commissioning local artists to develop meaningful and engaging logos or artwork (e.g., Dickerson et al., 2016; Venner et al., 2008). For others, it involved the inclusion of images depicting meaningful local places or community symbols (e.g., Linnaranta et al., 2024). For McNichols (2016), it was important that the original intervention’s images of predominantly White children be replaced to include Indigenous children.

##### 3.2.1.2 Language and metaphors

Many different language-related alterations were described. For one, some thought very intentionally about a culturally meaningful name for the adapted trauma intervention (e.g., Venner et al., 2008; Boyd-Ball, 2003). Others sought to remove academic jargon in the original text, thus making the adapted intervention easier for treatment providers to deliver and for clients to receive (e.g., Bedard-Gilligan et al., 2022; Pearson et al., 2019; Venner et al., 2008; Pearson et al., 2025). Others described changing the tone of the text to be more relatable (e.g., Campbell et al., 2023). For instance, Venner et al. (2008) described using a “Native voice (p. 22).” Venner clarified that changing the manual to be in a “Native voice” included keeping the manual in first person, using vignettes of Indigenous cases and quotes from Indigenous peoples, as well as inserting humor, where possible. One research team spoke on creating more opportunities for non-verbal forms of communication to accommodate cultural groups who rely more heavily on this mode (Linnaranta et al., 2024). More remarked on the efforts made to be mindful of and eliminate any language that focused on deficits and not resilience [disorders and disease state paradigms and terminology; (Linnaranta et al., 2024; Fox and Heitkamp, 2022; Pearson et al., 2020; Morsette et al., 2009)]. Further, some described relying on language experts to translate both the language of the intervention (either fully or select key words), as well as to better align metaphors and symbolism to a specific Indigenous community (e.g., Linnaranta et al., 2024; Campbell et al., 2023; Pearson et al., 2020; Morsette et al., 2009).

##### 3.2.1.3 Environment

Key parties rarely spoke on how an intervention’s conditions or surroundings were adapted to meet the cultural needs of Indigenous clients. The few that did speak to this surface-level adaptation mentioned ensuring the presence of sacred medicines like sweetgrass and sage within therapeutic spaces to make clients feel safe and welcome (e.g., Gray and Rose, 2012). There was also mention of displaying Indigenous art within therapeutic spaces (e.g., Gray and Rose, 2012). That is, including items like paintings and beadwork to communicate respect and appreciation. Others offered gifts to clients at some point over the therapeutic process as a gesture of respect and gratitude (e.g., Marsh et al., 2015b). For instance, Bedard-Gilligan et al. (2022) provided clients of their adapted program gift bags which included materials for their tribes’ specific cultural rituals. Lastly, the inclusion of food was an important element for some adapted projects. For instance, within Marsh et al.’s (2015a) adapted work, traditional feasts were held to mark the start and end of their several week-long therapy program. And for Ashby et al. (1988), meal sharing served as a central element of each of their adapted group counselling sessions.

##### 3.2.1.4 Content

The acknowledgement or inclusion of traditions or spiritual practices central to the Indigenous groups/clients being served were among the most frequently cited surface-level adaptations discussed. That is, common traditional practices incorporated within adapted trauma interventions included: sweat lodge ceremonies (e.g., Saylors, 2003; Hirchak et al., 2023; Marsh et al., 2015a; Wright et al., 2011); smudging (e.g., Pearson et al., 2019; Marsh et al., 2015a; Brave Heart et al., 2020; Heilbron and Gutrman, 2000); drumming (e.g., Saylors, 2003; Marsh et al., 2015a); pow-wows (e.g., Saylors, 2003; Marsh et al., 2015b; Brave Heart et al., 2020; Wright et al., 2011); singing (e.g., Saylors, 2003; Marsh et al., 2015b; Brave Heart et al., 2020); sharing circles [also referred to as healing and/or talking circles; (e.g., Edwards, 2003; Kelley et al., 2017; Brave Heart et al., 2020; Thomas and Bellefeuille, 2006)]; and praying (e.g., Heilbron and Gutrman, 2000; Morsette et al., 2009; Wright et al., 2011). And with inclusion of said practices, came a recognition that there are not always providers of an Indigenous cultural background available to lead these activities with clients. To supplement non-Indigenous providers’ lack of knowledge, some key parties and CRCs created guidance/tools within adapted intervention materials explicitly connecting selected traditions and spiritual practices to the original Western interventions (e.g., Venner et al., 2008; Bigfoot and Schmidt, 2010). That is, how does the traditional or spiritual practice complement the original Western treatment or healing model, and how does the traditional practice support healing in its own right?

Nevertheless, there also comes acknowledgement and appreciation from several key parties that some cultural traditions/spiritual practices are not for Western ears or eyes (e.g., Venner et al., 2008; Gray and Rose, 2012; Wendt et al., 2022). Therefore, some Indigenous traditions are meant to remain private and cannot be facilitated or witnessed by Western providers. In response to this concern, select key parties intentionally opted to include traditional practices and ceremonies that would not require the ceremony facilitator to have any sort of special status or Indigeneity (e.g., Novins et al., 2012). Others chose to refer clients outside of the adapted intervention for appropriate local Indigenous traditional, spiritual, and cultural supports, when needed (Bedard-Gilligan et al., 2022; Goodkind et al., 2010).

On a final note, while not described in great depth, there was mention of the inclusion of local history, allegories, and stories to adapted trauma interventions (e.g., Goodkind et al., 2010; Morsette et al., 2009). For example, Gray et al. (2010) described how several community Elders they worked in collaboration with wrote or told personal stories to be included as part of the team’s adapted program curriculum.

##### 3.2.1.5 Persons

In terms of intentional changes to those delivering the adapted trauma interventions, there was disagreement as to who was most appropriate. That is, some key parties were observed as believing that adapted treatments should primarily be delivered by Indigenous mental health providers, healers, or community members (e.g., McDonell et al., 2016; Hirchak et al., 2022). In response, when a mental health provider/team lacks Indigenous expertise or appropriate cultural knowledge (i.e., perhaps they identify as belonging to a vastly different Indigenous community than the one they are adapting the treatment for), some teams opted to partner or hire Indigenous healers/experts. That is, to supplement Western activities with traditional ones, assist in the processing of cultural experiences, or facilitate intervention delivery as a whole (e.g., Motta-Ochoa et al., 2026; Beckstead et al., 2015; Morsette et al., 2012). Of note, in such cases where feasibility and acceptance outcomes were measured, the involvement of Elders within therapy delivery or as available supports for clients was often remarked on as a strength of said adapted intervention by the program’s providers and clients (e.g., Marsh et al., 2016; Morsette et al., 2012).

Others acknowledged there is sometimes a lack of available individuals, with the appropriate community membership and the necessary trauma/mental health expertise, to facilitate delivery of adapted interventions. To fill this gap, some teams strove to increase the capacity of Indigenous communities to support their own people’s care. For example, when Brave Heart et al. (2020) worked to adapt a group model of interpersonal psychotherapy for trauma and grief, they paired senior treatment providers (Ph.D level, licensed psychologists, social workers, and nurses) with less experienced Indigenous providers (students and community providers) to cofacilitate their program’s group sessions. In such cases, non-Indigenous clinicians/researchers sometimes served as trainers and supervisors, offering regular consultation (e.g., Marsh et al., 2020).

Moreover, when Indigenous providers supported delivery of the adapted interventions, whether internally trained or externally recruited, select key parties mentioned vicarious traumatization (e.g., Marsh et al., 2020). Specifically, given Indigenous peoples exposure to similar sources of traumatic stress, some Indigenous providers may be at greater risk of more deeply relating to Indigenous clients’ pain/grief and having resulting negative psychosocial symptoms. Baking education, support, and supervision into the adaptation project were all cited as being helpful in preventing and minimizing the impacts of vicarious traumatization (e.g., Marsh et al., 2020).

#### 3.2.2 Deep level adaptations

As previously described, deep level changes are those that imbue a culture’s values, worldviews, and beliefs into an intervention.

##### 3.2.2.1 Goals

On the matter of alterations to an intervention’s goals so as to be more culturally relevant, key parties mostly described moving away from Western medical model’s typical fixation on psychopathology, towards a more expanded understanding of health and wellbeing. That is, Western-based interventions are often characterized as “medicalocentric,” “disorder-centric” and “reductionistic” (Fox and Heitkamp, 2022; Wendt et al., 2022). They focus largely on the neurobiological nature of mental disorders at the individual level and often fail to consider the historical, cultural and sociopolitical contexts impacting individuals and communities. Conversely, key parties adapting trauma interventions to meet the cultural needs of Indigenous clients, commented on adopting a definition of mental health that is much more holistic, and thus encompassing of physical, mental, emotional, and spiritual dimensions (e.g., Hirchak et al., 2023; Bigfoot and Schmidt, 2010; Fox and Heitkamp, 2022). For instance, in the adaptation of cognitive processing therapy van Stolk-Cooke and group described the addition of a pretreatment session aimed at grounding client healing to self, family, community, and spirituality (van Stolk-Cooke et al., 2025). Key parties also spoke of more intentionally moving clients towards a sense of empowerment and inner strength. As an example, consider how a research team worked with Inuit in Nunavik to adapt the goals of a cognitive behavioral therapy program which incorporated virtual reality (Linnaranta et al., 2024). In so doing, they purposefully shifted the treatment away from its original focus on PTSD symptoms. The team did not want participants to be assessed for traumatic event exposure or receive PTSD diagnoses. Instead, they considered it to be more culturally safe to focus broadly on developing individuals’ emotion regulation skills. Although no longer obviously trauma-focused under a Western-paradigm, the research team asserted that strengthening individuals’ emotion regulation skills would have net positive effects on clients’ wellbeing and resilience, while also decreasing maladaptive coping behaviors and trauma symptoms.

Another key goal of adapted treatments described by key parties was reconnecting or strengthening Indigenous individuals’ cultural identities (e.g., Venner et al., 2008; Marsh et al., 2015a; Boyd-Ball, 2003). Specifically, key parties not only commented on the healing effects of reclaiming connection with one’s Indigenous roots as well as having pride in one’s Indigeneity, but also on the strength of cultural identity as a protective factor against future stress and disease (e.g., Bedard-Gilligan et al., 2022). As a last element, select key parties discussed adjusting adapted intervention goals so that they were not only targeting individual healing, but also community healing (e.g., Heilbron and Gutrman, 2000).

##### 3.2.2.2 Context

Two contextual elements commonly made an appearance within the adapted trauma interventions explored. The first was the inclusion of content acknowledging, educating, and/or addressing the topic of historical trauma (e.g., Pearson et al., 2019; Hirchak et al., 2023; Fox and Heitkamp, 2022; Pearson et al., 2020). Recall, historical trauma describes the enduring suffering amassed in one’s own life span, but also across generations, as a function of settler colonialism. For examples of how the context of historical trauma was included in adapted interventions, first consider Bedard-Gilligan et al. (2022). In adaptation of NET, treatment providers and clients were coached on how to include historical trauma as a memory on their lifelines. That is, they were instructed to recall and capture memories of first learning about the traumatic experiences of relatives and ancestors. This experience was then recorded using first-person language so as not to violate NET core principles of including only autobiographical memories in one’s personal lifeline. By way of another example, Brave Heart et al. (2020) married a group-based IPT approach with the *Historical Trauma and Unresolved Grief Intervention* (HTUG). Brave Heart explained how IPT’s key concept of the “sick role” (a social role one plays when possessing a mental illness) was reframed using HTUG. Specifically, that the client’s symptoms of PTSD and complicated grief were explained in the context of “collective and generational traumatic events in which personal or idiosyncratic trauma are embedded and linked (p. 5).” They also shared that the team opted for an alternative name for this role given “sick” may be seen as victim blaming by Indigenous clients. Of note, while characterized as an essential element to healing in Indigenous communities by majority of key parties, some cautioned against the “medicalization of historical trauma (Wendt et al., 2022, p. 16).” Specifically, changing this construct, which is meant to be understood as and healed collectively, to something rooted and treated exclusively in the individual.

Another contextual element commonly discussed was individual and community resilience or survivance (e.g., Hirchak et al., 2023; Linnaranta et al., 2024; Bryant-Davis, 2019). Specifically, highlighting where possible the active role Indigenous peoples played in resisting settler colonialism, and in many ways thriving despite its oppressive forces. Little to no detail or examples were provided as to what this looked like on a project-by-project level.

##### 3.2.2.3 Concepts

The incorporation of values was a common concept or idea leveraged within several adapted intervention projects. For some, there was an acknowledgement that traditional values vary from community to community (e.g., Gray and Rose, 2012). Others operated under an assumption that there are certain values that can be incorporated or emphasized within adapted works that are generalizable across most Indigenous groups (i.e., are pan-Indigenous) [(e.g., Venner et al., 2008)]. For example, key parties commonly commented on and leveraged that Indigenous peoples are relational beings. That is, that individuals are interconnected to social (family, community, society) and natural (animals, plants) worlds. It was, therefore, not uncommon to see expanded definitions of client support circles, which saw family, extended family, and non-blood relatives participating in the therapeutic process (e.g., Tsosie et al., 2011; Hirchak et al., 2023; Novins et al., 2012; Venner et al., 2008). As seen within Bigfoot and Schmidt (2010) Indigenous peoples’ relational view also included connection to the spiritual world. However, a word of caution from Gray and Rose (2012), that Indigenous and Western understandings of specific values can at times differ. For instance, the value of “cooperation” in Western cultures is generally task-oriented, whereas within Indigenous cultures it relates more to social agreement, respect, and noninterference (Gray and Rose, 2012).

On another note, several key parties chose to anchor their adapted treatments (and sometimes the adaptation process itself) to the sacred symbol/teaching tool of the Medicine Wheel (e.g., Kelley et al., 2017; Novins et al., 2012; Walker et al., 2023; Marsh et al., 2015a; Bigfoot and Schmidt, 2010; Fox and Heitkamp, 2022). This circular symbol is broken into four quadrants or directions, each interdependent, and an equally necessary element for wellbeing (Mashford-Pringle and Shawanda, 2023). While this tool can be used in a variety of contexts, within a mental health context it typically asserts that one’s physical, mental, emotional, and spiritual domains be equally developed and balanced to be healthy. As a case, Dickerson et al. (2016) adapted motivational interviewing to be used with urban Indigenous youth experiencing alcohol-related difficulties. The adapted intervention consisted of three workshops, each beginning with a discussion of the Medicine Wheel. For example, in the third workshop, which rested more within the spiritual domain of the wheel, clients partook in an interactive discussion on how the choices they make about their alcohol use can impact their spiritual well-being. Clients were also invited to partake in a traditional prayer and sage ceremony.

### 3.3 The implementation stage

#### 3.3.1 Client fit

Once an adapted program is created and ready for testing or use with clients, several key parties commented on assuring appropriate client fit. Specifically, key parties made the point that each nation/tribe has their own unique histories of colonization which may influence how federal policies of assimilation have impacted a client’s/community’s cultural connectedness with their Indigeneity (e.g., Venner et al., 2008; Martin, 2013; Gray and Rose, 2012). As such, one’s Indigeneity does not automatically translate to their being served best by an adapted trauma treatment. Therefore, key parties comment on assessing clients’ readiness and desire to work with traditional (and/or Western) ways of healing [(e.g., Saylors, 2003)]. To support in this assessment, Bigfoot and Schmidt (2010) utilized the *American Indian/Alaska Native Affiliation Model*. The research team used this tool in collaboration with individuals and their families to identify clients with high/strong Indigenous affiliation (candidates for the culturally adapted treatment) and those with limited/no Indigenous affiliation (candidates for the original Western treatment). Similarly, McDonell et al. (2016) administered the *American Indian Enculturation Scale* (Winterowd et al., 2008), a 17-item measure which asks individuals about how often they participate in certain cultural activities (seek help from Elders, eat or cook Indian food, attend pow-wows). Responses are rated on a 7-point scale, where 1 is “not at all” and 7 is “a great deal”.

Other key parties commented on how cultural connectedness, and therefore client needs, can change over the course of treatment (e.g., Venner et al., 2008). As such, it was suggested that clients be assessed semi-regularly. More still spoke to a wariness of perpetuating assumptions of “cultural essentialism (Wendt et al., 2022, p. 15).” That is, to avoid drawing sharp lines or binaries between Indigenous and non-Indigenous perspectives. Accepting that Indigeneity is one aspect of someone’s identity (gender, sexual orientation, religion). Also, acknowledging that many individuals do not identify with just one culture (i.e., are bicultural or multicultural). Somewhat relatedly, select key parties highlighted cultural connectedness is not always neatly predictive of client service preferences. In a study that compared the engagement of Indigenous adults participating in original *Alcoholics Anonymous* (AA) programming and culturally adapted versions, researchers noted that cultural identity was not predictive of the type of meetings attended (Tonigan et al., 2020). That is, acculturation and enculturation measures were administered. And generally, urban Indigenous participants with higher enculturation (i.e., greater Indigenous cultural connectedness) were reported to be just as likely to attend mainstream AA programs as compared to Indigenous participants with higher acculturation (i.e., less Indigenous cultural connectedness) scores. In some settings, like at *Anishnawbe Health Toronto* health centre as well as *Village Sobriety Project*, where several Western and Indigenous healing service offerings exist and flexibility is possible, individual clients are provided the freedom to choose which combination of services they wish to partake in Reeves and Stewart (2014) and Mills (2003).

#### 3.3.2 Testing and outcome monitoring

One of the supposed benefits of a culturally adapted approach is that in starting with a Western trauma treatment, “there exists a prior evidence base which enhances the likelihood of treatment effectiveness for other populations (Sansone et al., 2022, p. 34).” While some may interpret this to mean no additional testing of the cultural adaptation is necessary, most disagree. As Rousseau and Kirmayer affirm, “the cultural adaptation of manual-based interventions should be encouraged, but it must be supported by a careful evaluation of the active role of the added components. Cultural adaptation may thus be seen as the merger of different strong therapeutic traditions, which potentiate each other, rather than as simply a cosmetic packaging and marketing of mainstream knowledge (Rousseau and Kirmayer, 2009, p. 954).” This belief in testing is evidenced by the sheer number of key parties who have committed to building an additional evidence base for their adapted intervention.

Testing of adapted trauma interventions tended to happen on a smaller scale (i.e., with pilot studies). Very few key parties utilized a randomized controlled trial (RCT), which are commonly understood to be the gold standard of research designs for evaluating the effectiveness of treatments (e.g., Pearson et al., 2019; Burduli et al., 2018; D’Amico et al., 2020; Campbell et al., 2023; Pearson et al., 2025). As Gone highlighted, when in an Indigenous community setting (which was commonly the case) it can be difficult to amass large sample sizes and engage in the necessary follow up assessments (Gone, 2023). Also, for some key parties it is deemed concerning, “unacceptable,” and “unfair” for a subgroup of the population tested to receive culturally relevant treatment and not others – making the randomization required for rigorous testing challenging (e.g., D’Amico et al., 2020; Hirchak et al., 2022; Morsette et al., 2012). Building an evidence base also takes time, and some communities’ needs were so great that key parties could not wait. Therefore, select teams released their adapted trauma treatments earlier than intended. For instance, Horn et al. (2008) worked in collaboration with a community for the adaptation of their smoking cessation program. Said community pushed for release of the adapted program while it was in draft form. Of their decision, Horn and team asserted “real world application can inform research” and “community based participatory research is scientific (p. 61 & 63).” Also valuing real world evidence generation: when three schools were originally earmarked for testing of an adapted *Cognitive Behavioral Intervention for Trauma in Schools*, Goodkind et al. (2010) decided to “practice” implementation in one of the three schools, further adapt the program as needed, and then launch in the remaining schools.

On the matter of outcomes, key parties typically used Western-based, non-culturally adapted outcome monitoring tools to determine if the adapted interventions were a success. Said Western tools were quantitative in nature, and often monitored change in PTSD, substance use, or trauma-adjacent symptom frequency or severity over time (e.g., Pearson et al., 2019; Venner et al., 2016; Tsosie et al., 2011; Goodkind et al., 2010; Gray et al., 2010; Tonigan et al., 2020). In other cases, key parties ensured the Western tools they used had been normed within Indigenous samples (e.g., Beckstead et al., 2015) or they modified item wording slightly so as to be more relevant/appropriate (e.g., D’Amico et al., 2020; Morsette et al., 2009). On rare occasions, key parties employed measures created specifically with Indigenous populations in mind. For instance, select research teams used the *Historical Loss Scale* and the *Historical Loss Associated Symptoms Scale* (Whitbeck et al., 2004) which assessed the frequency at which an individual thought about perceived historical losses as well as their emotional responses to these losses, respectively (McDonell et al., 2016; Brave Heart et al., 2020).

Gone commented on the aforementioned quantitative focus: there are “epistemological divergencies between knowing if Western and culturally sensitive therapies have worked (Gone, 2009, p. 759).” That is Western evidence-based therapies use scientific methods to test efficacy, which cannot measure/account for the spiritual nature of healing and generally devalue qualitative data. On the latter element Gone goes on to say that within Indigenous communities, the highest standard of a treatment’s efficacy is often the personal experience of its treatment providers and clients. Whether to consciously meet this standard or not, many key parties included a qualitative component as an element of their implementation process, probing treatment providers and clients as to their perception of the adapted treatment (e.g., Bedard-Gilligan et al., 2022; Thomas and Bellefeuille, 2006; Marsh et al., 2020). More specifically, “consumers’” overall satisfaction, likes and dislikes, perceived benefits, and suggestions for future improvement, among other things.

Within the academic and non-academic literature reviewed, efforts and calls were made to collect more culturally relevant outcomes for Indigenous peoples. For example, Ferguson (2023) compiled outcomes of culturally relevant trauma interventions for Indigenous peoples within Canada, and noted: “strengthened sense of connection to Indigenous identity; increased understanding of historical trauma; improved sense of empowerment and self-determination; spirituality; increased trust towards mental health providers, healers and community members, as well as trust in the self; improved relationality (i.e., connection to the land, other living things, each other; slide 14).” By way of another example, Edwards (2003) sought to measure the success of a culturally adapted 90-day residential substance use treatment program by understanding the number and quality of related transformational/healing experiences had by clients while in the program. They defined transformation experience as “any experience that made an important difference in the client’s mental, spiritual, emotional, or physical wellbeing (p. 54)”.

Others spoke of the value of engaging in strengths-based outcome monitoring. For instance, Goodkind et al. (2010) worked with their CRC to ensure measures were appropriate, including reviewing all individual items. The research team also decided to include a measure of children’s coping strategies to highlight strengths-based competencies. And finally, some key parties emphasized the importance of collecting individual and community wellness outcomes (e.g., Novins et al., 2012). For example, Kanate et al. (2015) combined Indigenous healing practices with opioid substitution therapy and buprenorphine-naloxone in working with an Indigenous community in Northwestern Ontario. In their data analyses, the team considered annual community-wide wellness outcomes like number of child protective cases, school attendance, police criminal charges, needle distribution, seasonal flu immunizations, etc.

## 4 Discussion

Although cultural adaptation represents only one approach to supporting Indigenous healing from contemporary and historical trauma, and may not be appropriate/desired in all contexts, this environmental scan suggests that it has been used with some frequency across Turtle Island. That is, going back some 40 years, key parties have sought to meet the unique needs of Indigenous clients by adapting Western trauma treatments so as to be more culturally relevant. From a bird’s eye view, many of these adaptation efforts share overarching themes. At the early planning stages, multidisciplinary expert and community engagement seemed vital, and key parties sometimes took additional steps to ensure they identified, understood, and appropriately described the “problem” they are trying to solve or improve. Attention was also often paid to ensure the proposed Western intervention’s alignment with Indigenous community values and priorities.

In the later adaptation phases, key parties could be seen employing a number of surface level adaptations through changes to the originating interventions’ visuals, language, conditions/surroundings, content, and personnel. Deep level adaptations were also common, with major shifts observed in conceptualizations of health/wellness goals, as well as the contextual learnings about Indigenous peoples’ collective soul wound and survivance which were deemed by some as necessary for healing. We also saw efforts to embed traditional values and sacred symbols/teaching tools within adapted works. And finally, within the end stages of adaptation, we saw the varied methods used to determine if clients were likely to benefit from an adapted treatment, or if they were in fact better served by the original treatment. We also observed within this implementation phase the diverse research design and data collection methods, with a great deal of focus on including more appropriate outcomes for culturally adapted trauma treatments.

Yet, once our feet land firmly on the ground, we see gross variability emerges on a project-by-project basis. This diversity in the adaptation process is in part likely owed to the creativity and flexibility required by key parties to meet the unique cultural needs of the distinct community(ies) they are working with. That is, while many (including us, the authors within this article) sometimes refer to Indigenous peoples as though they are a monolith, they are very much heterogeneous groups with varied cultures, traditions, histories, and ways of knowing. In combination with the absence of formal guidelines on how to go about the intervention process within this population, extreme differences between adaptation projects are to be expected.

Taken together, these observations point to a more fundamental uncertainty within the current landscape. In the absence of formal guidance and a sufficient centering of Indigenous knowledge holders (and other appropriately situated voices) informing said guidance, the fact that cultural adaptation is happening should not be taken as evidence that it is necessarily wanted, needed, or a sufficient response to the limitations of Western trauma care. Rather, there remains a need to ask whether cultural adaptation is viewed as a meaningful pathway to healing. And if so, for whom, when, and under what conditions. To meet this need we propose an intentional, respectful, and transparent conversation between Indigenous and non-Indigenous knowledge holders. This conversation should include Elders, healers, community leaders, mental health providers, scholars, and people with lived experience. And if cultural adaptation is viewed a potentially valuable option, these same voices will be key in helping to identify what is needed for such efforts to be culturally appropriate, meaningful, valued, safe and effective. In summary, Wise Practices outlining what is needed to minimize the risk for adaptation to reproduce the very harm and trauma it is seeking to address. We aim to serve as facilitators for this important dialogue in the next part of this series.

Also worth commenting on, for those interested in further exploring the current cultural adaptation landscape of Western trauma interventions for use within this population, there may be benefit to engaging in additional cross analyses. Specifically exploring: Are there differences in cultural adaptation practices between Canada and the United States? Are there differences between current cultural adaptation practices in Western Canada (typically seen as taking the lead in decolonization of education, healthcare and social services) versus Eastern Canada? Has there been an evolution in certain adaptation patterns of thought over time? And are certain Indigenous communities or groups making greater use of culturally adapted services than others? On expanding the scope of the project, additional research could be done to investigate: What are the similarities and differences in the cultural adaptation of treatment interventions versus Western prevention and promotion initiatives? Further, are there similarities and differences between cultural adaptation intervention practices for trauma treatments as compared with other types of difficulties (psychosis, mood disorders, anxiety)?

### 4.1 Strengths and limitations

Several strengths of this environmental scan should be noted. First, we (the authors) attempted to explore the aforementioned research questions using a decolonial lens. Meaning, Indigenous-specific databases and non-academic literature were included to broaden the forms of knowledge and practice visible within the scan. In so doing, we also sought to afford due respect and consideration to knowledge generated and shared outside conventional academic publishing systems. Although few grey literature sources were ultimately identified that described the cultural adaptation efforts of Western trauma interventions, especially relative to more culturally grounded approaches, their inclusion remained vital. Second, the broader conceptualization of trauma used within this scan was also a strength as it helped to decenter Western, often individualistic and pathologizing understandings of trauma. In its place, greater space was made for culturally-situated understandings of distress, healing, and resilience. Third, despite being designed as an environmental scan rather than a systematic review, this search was undertaken in a rigorous, structured, and transparent manner. That is, it used predefined search terms and eligibility criteria, multiple source streams, and was reviewed by specialist librarians. Finally, this scan focused on *how* Western interventions are being culturally adapted, rather than whether these adaptations are effective. This unique focus makes an often elusive and glossed-over aspect of the broader field of Indigenous mental health and healing more visible, offering a starting point for critical and constructive reflection by Indigenous and allied knowledge holders.

Nevertheless, this study has several limitations that must be acknowledged, which may affect the interpretation and generalizability of the findings. They can be categorized into methodological, structural, research team, and knowledge translation concerns.

#### 4.1.1 Methodological limitations

A significant limitation of this article arises from potential sampling bias and representation gaps. Our reliance on published literature and online content may overlook community-based, unpublished, or orally transmitted adaptation efforts that are crucial for understanding the full landscape of Indigenous adaptation initiatives. Additionally, there may be an overrepresentation of larger, more well-funded programs with the resources necessary for documentation and evaluation, while rural, remote, or smaller Indigenous communities may be underrepresented. This discrepancy limits our understanding of the diverse adaptive strategies employed across different contexts.

Additionally, the environmental scan solely focuses on English-language publications and resources, likely excluding valuable adaptations documented in Indigenous languages or in French (Canada’s second official language), thereby narrowing the scope of our analysis and possibly perpetuating inequalities in available knowledge. There also exists a tendency to prioritize the publication of successful adaptations, with unsuccessful attempts often undocumented. This aspect of academic publishing, combined with institutional barriers often faced by marginalized groups, further complicates our ability to access and include timely information about innovative practices undertaken to support Indigenous clients and communities.

#### 4.1.2 Structural limitations

It is also important to remark on the fact that this environmental scan primarily focuses on adapting Western interventions rather than centering Indigenous healing systems. This approach may inadvertently perpetuate the notion that Western methodologies are the normative standard to be modified, overshadowing the diversity and validity of Indigenous systems. However, it is essential to acknowledge that much of what Western science claims as “original” may have roots in Indigenous paradigms, highlighting the need for greater recognition of these foundational contributions. Moreover, Indigenous Peoples have historically healed and sustained themselves through their own robust healing practices, which have not only been effective for their communities over generations but have also informed and enriched the healing practices of Western populations. Notably, Western societies have benefited from many Indigenous healing approaches and paradigms. For instance, consider psychedelic-assisted psychotherapy and various plant-based medicines, both in the past and in contemporary contexts (Williams et al., 2022). We also want to emphasize that culturally grounded treatments should be regarded as a recommended first-line option whenever feasible and appropriate for clients and their specific settings. Acknowledging and respecting the wisdom of Indigenous healing systems not only enhances our understanding of trauma and their potential interventions but also underscores the importance of collaborative and inclusive approaches in addressing health disparities.

The lack of cultural specificity in this environmental scan is another concern, as it risks oversimplifying the rich tapestry of Indigenous cultures across Turtle Island. By generalizing across cultures, this review may contribute to harmful notions of cultural competence that imply: (i) Indigenous cultures are homogeneous, (ii) a universal standard exists for what works for all Indigenous peoples, and (iii) education is merely a utilitarian means to an end. It is essential for readers to understand that this work represents only an initial step in a continuous learning process. The general nature of our findings underscores the necessity for further collaboration with Indigenous communities to obtain deeper insights and context.

#### 4.1.3 Research team limitations

The involvement of only one Indigenous collaborator highlights another limitation of this study, as it is unlikely to ensure the review reflects the vast diversity of Indigenous cultures. The onus of ensuring cultural safety and accountability often fell disproportionately on this individual, which may lead to potential biases in this environmental scan’s methodology and presentation. Moreover, while this research is framed as collaborative, it is not community driven. A more effective approach might have included establishing trust and relationships with local Indigenous communities from the outset and employing a community-based participatory research framework. Such an approach would enhance specificity and reduce the reliance on Western paradigms. Ultimately, we opted against this approach in order to educate ourselves first, given the significant burden already placed on Indigenous experts in similar contexts. Moreover, now having educated ourselves on the cultural adaptation landscape, the plan for the next phase of this research is to formally consult and engage Indigenous and non-Indigenous advisors on this topic. That is, better understand key informants’ perceptions of culturally adapted Western trauma interventions as a tool for healing with Indigenous populations *and* to understand what steps are needed to engage in the process of cultural adaptation safely and effectively. The work continues.

#### 4.1.4 Knowledge translation concerns

Our environmental scan provides a static snapshot of adaptations that are dynamic and evolving. Compounding this issue, it appears that most key parties engaged in a “one-and-done” approach: they tended to adapt an intervention, conduct initial testing, and then move on without further modifications or subsequent evaluations. This lack of ongoing inquiry means that few key parties maintained a sustained commitment to a single treatment or continued to track its effectiveness over multiple iterations of adaptations. This limitation presents challenges in accurately representing adaptations that respond to changing community or population needs, possibly leading to misinterpretations of their relevance and impact. To truly understand the effectiveness and adaptability of these interventions, a more comprehensive and long-term approach to evaluation is necessary, one that recognizes the fluidity of community dynamics and the importance of iterative learning.

The nature of this scan is primarily applied, focusing heavily on the ‘how’ of adaptations rather than assessing their effectiveness or outcomes. However, many adaptations relevant to our research lacked detailed descriptions of the processes involved, with key parties more inclined to focus on the outcomes or success of their endeavors rather than the methods employed. This lack of detailed insight leaves gaps in understanding the full context and complexity of adaptation efforts.

## Footnotes

1 Within Indigenous scholarship, to be called a “Settler” means that you are non-Indigenous and that either you or your ancestors willingly came to reside on land inhabited by Indigenous peoples (Thomas, 2019). As such, those that have come to reside on Turtle Island as a function of slavery, or as refugees displaced from their home countries due to violence, conflict or persecution, do not typically fall under the term.

2 Note, the authors in no way wish to perpetuate the idea that Indigenous people are unwell but rather are culturally rich peoples who were victims to settler colonial practices and ethnocide. Turtle Island’s Indigenous peoples are not sick, but they are suffering from physical, psychological, and spiritual symptoms born of a sick colonial system (Dupuis-Rossi, 2020; Nelson et al., 2014).

3 Within this work, the authors use Williams et al. (2021) synthesis of allyship to inform allyship goals. For instance, *allyship* is about enacting a continual process of self-reflection; engaging in coalition building and working in solidarity with those we wish to ally ourselves with; acting out of genuine care and concern for others; etc.
