# Supplementary material for: The cultural adaptation of Western trauma interventions for Indigenous peoples experiencing trauma: an environmental scan of Turtle Island
Source: Front Psychol. 2026 Oct 29;17:1927776. doi: 10.3389/fpsyg.2026.1927776 (PMC13755588; doi:10.3389/fpsyg.2026.1927776)
Supplement: Supplementary file 1 [file Data_Sheet_1.PDF]

Supplementary Table A. ***Intervention-specific cultural adaptation sample discussed organized by year.***

Year	Author(s)	Location	Clinical Population Involved	Clinical Focus	Western Intervention Adapted
1988	Ashby et al.	United States	American Indian adolescent girls	trauma (sexual abuse)	group treatment (unnamed)
2000	Heilbron & Guttman	Canada (inferred)	Ojibway First Nations women; on reservation	trauma (child sexual abuse)	Cognitive Therapy
2003	Boyd-Ball	United States	American Indian and Alaskan Native adolescents and their families; multiple tribal/community affiliations	alcohol/substance use	Family Check-Up
2003	Edwards	United States	Native American adults in residential treatment; urban setting	alcohol/substance use; trauma	Alcoholics Anonymous
2003	Mills	United States	Yup'ik and Cup'ik Alaskan Natives	mental health (general); substance use	multiple Western treatment modalities/techniques integrated (including Cognitive Therapy)
2003	Saylors	United States	Native American women; urban setting	mental health (general); substance use; trauma	mental health services generally
2006	Thomas & Bellefeuille	Canada	Aboriginal adults with residential school experience	mental health (general); trauma	Focusing technique
2007	Venner et al.	United States	Native American adults; multiple tribal/community affiliations	alcohol use	Motivational Interviewing
2008	Horn et al.	United States	American Indian adolescents	tobacco use	Not On Tobacco cessation program
2009	Dionne et al.	United States	families with American Indian children	parenting and child behaviour (trauma-conceptualized)	Incredible Years Parenting program
2009	Morsette et al.	United States	American Indian adolescents; rural reservation	depression; trauma (violence exposure)	Cognitive Behavioral Intervention for Trauma in Schools
2010	BigFoot & Schmidt	United States	American Indian and Alaskan Native children	trauma	Trauma-Focused Cognitive Behavioral Therapy
2010	Goodkind et al.	United States	American Indian adolescents from three Southwestern tribal communities; rural reservation	anxiety; depression; trauma (violence exposure)	Cognitive Behavioral Intervention for Trauma in Schools
2010	Gray et al.	United States	American Indian women from one Southwestern tribal community	alcohol use; depression	Cognitive-Behavioral Skills Building

2011	Tsosie et al.	United States	physically injured American Indian and Alaskan Native adults; multiple tribal/community affiliations	trauma (psychological stress from physical injury)	care management intervention based on a stepped collaborative care model
2011	Wright et al.	United States	American Indian and Alaskan Native adults in outpatient and residential treatment; urban setting	mental health (general); substance use	multiple Western treatment modalities/techniques integrated (including Motivational Interviewing)
2012	Martin	United States	Native American women; on reservation	trauma (historical)	Narrative Therapy
2012	Morsette et al.	United States	American Indian children and adolescents	depression; trauma (violence exposure)	Cognitive Behavioral Intervention for Trauma in Schools
2012	Novins et al.	United States	Native American adolescents and their families	substance use	Cognitive Behavioral Therapy + Contingency Management + Motivational Interviewing
2014	Reeves & Stewart	Canada	Indigenous clients; urban setting	trauma (sexual abuse)	mental health services generally
2015	Beckstead et al.	United States	American Indian and Alaskan Native adolescents in residential treatment; multiple tribal/community affiliations	mood difficulties; substance use	Dialectical Behavior Therapy
2015	Campbell et al.	United States	American Indian and Alaskan Native adults; urban setting	substance use	Community Reinforcement Approach
2015	Kanate et al.	Canada	North Caribou Lake First Nations adults; remote setting	opioid use	buprenorphine-naloxone substitution therapy with addictions counselling (unnamed)
2015a	Marsh et al.	Canada	Aboriginal peoples	substance use; trauma (intergenerational)	Seeking Safety
2015b	Marsh et al.	Canada	Aboriginal adults; urban setting	substance use; trauma (intergenerational)	Seeking Safety
2016	Dickerson et al.	United States	American Indian and Alaskan Native youth; urban setting	substance use	Motivational Interviewing
2016	Marsh et al.	Canada	Aboriginal adults; off-reserve	substance use; trauma (intergenerational)	Seeking Safety
2016	McDonell et al.	United States	American Indian and Alaskan Native adults; urban setting and rural reservation	alcohol use	Contingency Management
2016	McNichols	Canada	Lytton First Nations children/youth and their families	trauma (complex; intergenerational)	Complex Care and Intervention program

2016	Venner et al.	United States	American Indian and Alaskan Native adults from one Southwestern tribe; on/near reservation	substance use	Community Reinforcement Approach + Motivational Interviewing
2017	Kelley et al.	United States	American Indian adults; urban setting and reservation	substance use	Peer Recovery Support
2018	Burduli et al.	United States	American Indian adults on one Northern Plains reservation; on-reservation	alcohol/substance use	Contingency Management
2019	Pearson et al.	United States	Native American women; on/near rural reservation	high-risk sexual behaviour; substance use; trauma	Cognitive Processing Therapy
2020	Brave Heart et al.	United States	American Indian adults; urban and rural setting	depression; grief; trauma	Group Interpersonal Psychotherapy
2020	D'Amico et al.	United States	American Indian and Alaskan Native adolescents; urban setting	alcohol/substance use	Motivational Interviewing
2020	Marsh et al.	Canada	Indigenous adults	substance use; trauma (intergenerational)	Seeking Safety
2020	Pearson et al.	United States	Native American adolescents and adults; on/near reservation	high-risk sexual behaviour; substance use; trauma	Cognitive Behavioral Therapy + Motivational Interviewing vs Narrative Exposure Therapy
2020	Tonigan et al.	United States	American Indian adults; urban setting	alcohol use	Alcoholics Anonymous
2021	Venner et al.	United States	American Indian and Alaskan Native adults; rural reservation	substance use	Community Reinforcement Approach + Motivational Interviewing
2022	Bedard-Gilligan et al.	United States	American Indian adolescents and adults	trauma	Narrative Exposure Therapy
2022	Hirchak et al.	United States	American Indian and Alaskan Native adults	substance use	Community Reinforcement Approach + Motivational Interviewing
2023	Campbell et al.	United States	American Indian and Alaskan Native adults; urban setting	substance use	Community Reinforcement Approach
2023	Hirchak et al.	United States	American Indian and Alaskan Native adults; urban setting and reservation	opioid use	medications for opioid use disorder
2023	Walker et al.	United States	American Indian and Alaskan Native adults from one Northwest tribal community; rural setting	substance use	Cognitive Behavioral Therapy + Motivational Interviewing

2024	Linnaranta et al.	Canada	Nunavik Inuit	trauma	Cognitive Behavioral Therapy with Virtual Reality
2025	Pearson et al.	United States	American Indian and Alaskan Native adolescents and adults with a Pacific Northwest tribal community; rural setting	high-risk sexual behaviour; substance use; trauma	Cognitive Behavioral Therapy + Motivational Interviewing vs Narrative Exposure Therapy
2025	van Stolk-Cooke et al.	United States	American Indian and Alaskan Native women	high-risk sexual behaviour; substance use; trauma	Cognitive Processing Therapy
2026	Hirchak et al.	United States	American Indian emerging adults; rural reservation	alcohol use	Contingency Management
2026	Motta-Ochoa et al.	Canada	First Nations, Inuit, Metis adults; urban setting	alcohol use	Managed Alcohol Programs

Notes . This table summarizes cultural adaptation projects discussed in some depth within the present environmental scan and should not be interpreted as an exhaustive account of the literature. That is, some relevant work may not have been captured by the search strategy, while other interventions focused primarily on outcomes and less on adaptation processes, which is the focus of this scan. Additionally, some of the above articles represent multiple knowledge outputs from, or expansions of, the same project/body of work (e.g., Morsette et al., 2009, 2012; Goodkind et al., 2010).
